# Exploring Approaches for Detecting Protein Functional Similarity within an Orthology-based Framework

**DOI:** 10.1038/s41598-017-00465-5

**Published:** 2017-03-23

**Authors:** Christian X. Weichenberger, Antonia Palermo, Peter P. Pramstaller, Francisco S. Domingues

**Affiliations:** Center for Biomedicine, European Academy of Bozen/Bolzano (EURAC), (Affiliated to the University of Lübeck, Lübeck, Germany), Viale Druso 1, 39100 Bolzano, Italy

## Abstract

Protein functional similarity based on gene ontology (GO) annotations serves as a powerful tool when comparing proteins on a functional level in applications such as protein-protein interaction prediction, gene prioritization, and disease gene discovery. Functional similarity (FS) is usually quantified by combining the GO hierarchy with an annotation corpus that links genes and gene products to GO terms. One large group of algorithms involves calculation of GO term semantic similarity (SS) between all the terms annotating the two proteins, followed by a second step, described as “mixing strategy”, which involves combining the SS values to yield the final FS value. Due to the variability of protein annotation caused e.g. by annotation bias, this value cannot be reliably compared on an absolute scale. We therefore introduce a similarity z-score that takes into account the FS background distribution of each protein. For a selection of popular SS measures and mixing strategies we demonstrate moderate accuracy improvement when using z-scores in a benchmark that aims to separate orthologous cases from random gene pairs and discuss in this context the impact of annotation corpus choice. The approach has been implemented in Frela, a fast high-throughput public web server for protein FS calculation and interpretation.

## Introduction

Gene products can be compared in many different ways, researchers for example have been performing comparisons between proteins based on the amino acid similarity^1^ for many years. Proteins can further be computationally compared regarding their molecular function and biological role in the cell, provided there is a set of standardized annotations that can be used to calculate measures of semantic similarity^2^. The Gene Ontology (GO) Consortium has been making major contributions in this area by establishing a controlled vocabulary of defined terms for annotating gene and gene product properties across species^3^. More specifically, GO provides three independent ontologies to describe biological molecules: Molecular Function (MF), Biological Process (BP), and Cellular Component (CC). Starting as a collaborative annotation project between fruit fly, yeast, and mouse model organism databases, GO now contains a growing collection of ontology terms as well as a large amount of annotations from all kingdoms of life. GO terms are organized in a directed acyclic graph (DAG) with the so-called root term being the most common term with only child terms. More specific terms are further away from the root and any term (other than the root) may have multiple child and parent terms.

Soon after the introduction of GO, researchers started to apply semantic similarity concepts from natural language ontologies to GO-annotated gene products^4^, defining a function that maps a pair of GO terms to a numeric value expressing the semantic similarity (SS) of this pair. Now, more than a decade later, roughly 100 different methods have been suggested to compute SS for pairs or sets of GO terms^5^. Several reviews are available^6–9^, which attempt to classify the different methods into groups with related SS measures. Generally, these measures make use of the structure of the DAG and/or combine this with information from a GO annotation corpus, which provides the mapping between GO terms and gene products. Following the classification suggested by Guzzi *et al*.^7^, measures may be distinguished by their use of ancestor terms, whether they utilize DAG structural properties such as term depths or path lengths, or if they consider the term information content, which relates to negative logarithm of the probability to observe a GO term or a more specific term in the annotation corpus given the DAG. The SS measures have also been classified as pairwise or group-wise, according to how the multiple GO terms annotated to each protein are taken into account. In pairwise measures two proteins are compared semantically by either computing all possible pairwise semantic similarities between the GO terms annotated to each of the two proteins and determining a suitable combined score (often referred to as mixing strategy), for example by taking the maximum of all SS values. Alternatively, group-wise measures perform a direct comparison of sets of GO terms. Ultimately, some measures apply vector space models, where the GO terms annotated to a protein are encoded as a vector, and two vectors are then used to compute the SS of the respective proteins.

Protein semantic similarity has been applied to answer various biological questions, such as protein-protein interaction (PPI) prediction^10^, and conversely, prediction of false positives in PPI networks^11^, prediction of pathways from PPI genome-wide data^12^, imputation of missing values in expression data^13^, construction of SS networks to discover disease genes^14^, protein function prediction^15^, just to name some recent applications, see Pesquita *et al*. for a more complete overview^9^. Performance of SS measures was also benchmarked on PPI data and on their relationship to sequence similarity and gene expression data, summarized in the CESSM tool for assessment of SS measures^16^.

Over time, several groups identified and discussed drawbacks of individual SS measures and the annotation process as a whole. Evidently, protein annotation is biased and is influenced by different research interests, with model organisms of human disease for example being better annotated^17^ and promising gene products (e.g. disease associated genes) or specific gene families having a higher number of annotations. These biases have been analysed over time^18^ and lead to correlations between the number of GO terms a protein is annotated with, which in turn affects applications that involve SS measures^19^. Protein annotations with GO terms include evidence codes, which inform about the source of the annotations and which are frequently interpreted as a quality index. In particular, use of automated annotations (inferred from electronic annotation, IEA), which form the majority of all available annotations, has been investigated in most SS assessments. In general, these assessments report that including annotations with IEA evidence codes improves performance most of the time^7^, and it is encouraging to see electronic annotations improve over time^20^. Finally, certain SS measures are affected by shallow annotations, which are very generic/unspecific annotations close to the root, in the sense that two proteins furnished with only shallow annotations receive high SS scores^14, 21^.

Considerable effort has been put into developing software packages and web servers to compute SS, see reviews by Guzzi *et al*.^7^ and Gan *et al*.^6^, with subsequent work published as web services or extensions to existing web resources^22–25^ or as downloadable source code^26–29^. Notably, the GOssTo framework allows calculation of several popular SS measures both as a standalone Java program or through a web service^30^, whereas the Semantic Measures Library offers about 50 SS measures in a command line Java toolkit. Ultimately, Mazandu *et al*. provide the most complete set of SS measures both for online calculation^31^ and as a high-performance Python command line tool^5^.

Here, we present an orthology-based evaluation of different functional similarity (FS) measures, resulting from the combination of six popular SS measures with five mixing strategies. We tackle annotation bias by introducing protein-specific z-scores and discuss their performance when compared to conventional FS scores. This work also reinvestigates the influence of electronic annotations and annotation corpus choice on the performance of the FS measures with respect to all three available gene ontologies. We furthermore combine FS scores from the different ontologies and compare their performance. The methods can be run on a new web server, http://frela.eurac.edu, which allows high-throughput calculations on an average of several ten thousand protein pairs per minute. The server facilitates biological interpretation of computed FS scores. The software, including the source code for the web server, is available for download from our web server.

## Results and Discussion

In this work, we have compared the performance of selected functional similarity measures in discriminating pairs of orthologous genes from random pairs and utilized protein-based z-scores as a means to improve the discriminatory power of these FS measures. To summarize, we have chosen the following six pairwise SS measures: Resnik (*simRes*), Lin (*simLin*), Schlicker (*simRel*), information coefficient (*simIC*), Jiang and Conrath (*simJC*), and graph information content (*simGIC*). We combined these SS measures with each of the following five mixing strategies: average (*fsAvg*), maximum (*fsMax*), maximum of best matches (*fsBMM*), best matches averaged (*fsBMA*), and mean of best matches (*fsABM*), which gives a total of 30 FS measures that were investigated in our study.

All SS measures (except *simGIC*) discussed here make use of the most informative common ancestor (MICA), that is, the common ancestor of the two compared GO terms that has maximal information content. The earliest SS measure is *simRes*, which simply selects the information content of the MICA. This measure is not normalized and neglects any information about the contributing GO terms. Since *simRes* is not bounded from above, any normalization procedure (such as division by the highest information content value^32^) depends on the annotation corpus itself, and therefore hampers score comparison between corpora. To limit these drawbacks, *simLin* normalizes *simRes* by the sum of the contributing GO terms’ information contents, such that MICAs that are close to their GO terms receive a higher score than those that are higher up in the GO graph. In *simRel*, the level of annotation detail is addressed by weighting *simLin* with the counter-probability of the MICA. By this, shallow annotations receive less relevance than MICAs further away from the root. A criticism of this approach was that annotations close to the root or to the leaves receive too little weights, which was addressed by an alternative weighting scheme in the *simIC* measure. Initially designed as a metric^33^, then transformed into a SS measure^34^, *simJC* shows little difference to *simLin* ^35^. Ultimately, *simGIC* takes into account all ancestor terms and, most notably, does not make use of MICAs.

Two gene products are functionally compared by computing a semantic similarity score matrix filled with SS scores formed by pairing annotations made to one gene product versus the annotations to the other. The mixing strategies operate on the SS score matrix in order to obtain a single FS score. Each of the mixing strategies has been designed with a specific goal. When choosing *fsAvg* ^4^, a high score can only be obtained if all SS scores are high, i.e. if the two proteins are annotated with very similar terms. On the other hand, *fsMax* ^36^ highlights the largest SS score and thus points to the most similar subfunctionality. The remaining mixing strategies are variations of row and column maxima functions, which express the highest similarity of one specific annotation to all annotations of the comparison partner and vice versa. Historically, *fsABM* was mentioned first^37^, and was later discussed in detail as an improved mixing strategy^38^ by taking the average over all row and column maxima. Like *fsAvg*, this strategy works well if both proteins have many related annotations. The *fsBMA* ^34^ strategy computes the average of the averaged row and column maxima and therefore takes better into account individual high similarities, whereas *fsBMM* ^39^ puts emphasis on an asymmetric view by selecting the score obtained from the better average maximum, highlighting good partial matches in the respective protein.

### Annotation Bias and Score Distribution

**Figure 1:**
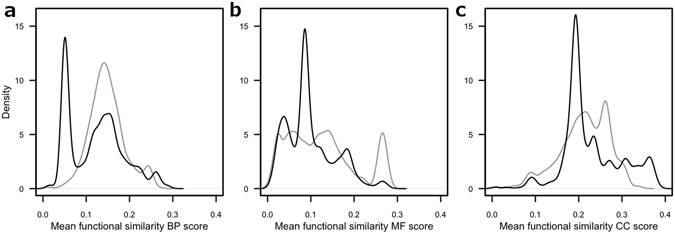
Average *simLin*/*fsAvg* score distributions for BP, MF, and CC ontologies for human/mouse protein pairs. For a human protein *P*, the score average is computed by forming pairs of proteins (*P*, *R*) over 1000 randomly selected mouse proteins *R*, with the kernel density estimates of the respective distributions being displayed for the IEA^(+)^ dataset (black solid lines, density computed from 93806 annotated proteins, also see Table 1) and the IEA^(−)^ dataset (grey lines, 21212 annotated proteins). (**a**) BP score distribution. Manually annotated protein pairs show a clear peak at a *simLin*/*fsAvg* score of 0.15. Including IEA evidence codes in the annotation corpus generates a second peak very close to 0.0. A large portion of this peak can be attributed to the roughly 70000 human gene products, which are exclusively annotated with IEA evidence codes. (**b**) MF based score distribution. Unlike BP with its sharp peak for the manual annotations, this ontology is characterized by a more uniform distribution of scores, with a notable peak near 0.27, generated by approximately 1600 proteins. GO enrichment analysis of these proteins shows that they are significantly enriched in “protein binding” (GO:0005155, *p* < 10^−100^), suggesting that gene products annotated to this term generally yield much higher than average *simLin*/*fsAvg* MF scores. (**c**) CC score distribution. Here, both manual and electronic annotation peaks are closer to each other than in the other two ontologies. Furthermore, electronic annotations are characterized by densities in the upper score range (>0.3), where the manual annotation scores have already tailed off.

Several studies have examined annotation bias in GO. Early on, Wang *et al*.^19^ have reported that heavily studied and annotated disease genes and their orthologues in model organisms are one of the sources of bias, and that this would be influencing gene prioritization results. The authors propose a correction by a power transformation for FS scores that uses parameters estimated from random background distributions of FS scores. Shallow annotations can be interpreted as the inverse of annotation bias, and Chen *et al*.^40^ suggested to overcome shallow annotations by calculating an information content overlap ratio FS score, which essentially is a reciprocal average of information content scores. This idea has been refined by Teng *et al*.^24^ by taking into account shared information content during score calculation, a concept that has been introduced earlier albeit tackled differently^34^. Motivated by the aforementioned findings of Wang *et al*.^19^, Schulz *et al*.^41^ introduced a method to calculate exact *p*-values for a fixed set of query terms and applied this concept for similarity searches in phenotype ontologies. A temporal analysis of annotation bias in GO database releases over ten years revealed an increase for human data, but also a decrease in yeast data^18^. Another source for annotation bias are high-throughput experiments, which contribute disproportionally large amounts of annotations by only few published studies^42^, being further propagated by automated methods. This huge body of electronic annotations (evidence code IEA) has a strong influence on scores. Figure 1 shows the distribution of score averages calculated from pairwise comparison of 1000 randomly chosen mouse proteins for each annotated human protein in the respective annotation corpus for the *simLin*/*fsAvg* measure. The scores are computed both when taking into account annotations with evidence code IEA (IEA^(+)^) and also when excluding IEA annotations (IEA^(−)^), see Table 1 for dataset sizes. In the distributions we find no single average score higher than 0.4, and since the averages were calculated over randomly selected protein pairs, this can conservatively be seen as a global upper limit for distinguishing random from non-random matches by *simLin*/*fsAvg* score. Each distribution exhibits a sharp peak in the lower score ranges that is absent in the case of scores calculated from non-electronic annotations. Since the scores are averages of proteins with randomly chosen partners, the peaks correspond to background similarity due to the electronic annotation process. This can be rather extreme, as for example is the case in the BP ontology (Fig. 1a), where in the presence of IEA annotations there are 13.4% scores below 0.05, but in their absence, this is reduced to 0.7%. The score distribution within the CC ontology (Fig. 1c) furthermore shows that IEA annotations can contribute substantially to obtaining higher scores, as the tail of the IEA^(+)^ distribution is elongated by almost 30% of the length of the IEA^(−)^ distribution. This tail accounts to proteins annotated with only IEA evidence codes.

**Table 1:** Number of annotated proteins in the organism data sets.

Ontology	Human^a^	Mouse^a^	Fly^a^
BP	93806 (21212)	16591 (14786)	11061 (9714)
MF	87067 (20155)	15594 (13503)	9165 (7960)
CC	84067 (22624)	17437 (15186)	8397 (7488)
BP+MF	78792 (17394)	14545 (12489)	8080 (6531)
BP+CC^b^	76002 (17397)	15432 (13054)	7610 (6603)
MF+CC^b^	66261 (15844)	14720 (12298)	6897 (5975)
BP+MF+CC	63584 (14814)	13979 (11598)	6571 (5528)

^a^Numbers refer to IEA^(+)^ data sets, whereas numbers in parentheses refer to IEA^(−)^ data sets. ^b^Available on the Frela web server, but not further discussed in this work.

As mentioned before, genes with a higher number of GO annotations tend to receive higher FS scores. In other words, genes with annotation bias introduced by a large number of GO annotations are expected to have on average higher FS scores. To address this, Konopka *et al*.^43^ suggested using as a similarity threshold the 95% quantile derived from scores calculated from random protein pairs. Alternatively, we propose to improve the similarity scores of two proteins by taking into account their respective score background distribution and calculate a similarity z-score (see Methods) that is less affected by annotation biases of specific proteins. This requires calculation of mean and standard deviation for each protein *P* by evaluating FS scores from protein pairs (*P*, *Q*), where proteins *Q* are randomly sampled. The so derived mean score for protein *P* represents a baseline score that varies from protein to protein (see Fig. 1), and describes the expected FS score found for random protein pairs, which in turn reflects (but is not limited to) annotation bias. Together with a protein-specific standard deviation this allows to transform a FS score into a normalized z-score, which adjusts for the annotation baseline of the proteins compared.

As a first example, we consider the orthologous genes encoding human and mouse alcohol dehydrogenase 1 (UniProt accession numbers (UPANr) P07327 and P00329, respectively), which have a *simLin*/*fsAvg* IEA^(+)^ score of 0.22 in BP ontology. A similar score is computed for the unrelated pair of a human G-protein coupled receptor and a mouse histone protein (UPANr Q96LA9 and Q8CGP5). However, the latter protein pair has an average *simLin*/*fsAvg* score of 0.21 for 1000 randomly chosen mouse proteins, and in this context the particular score of 0.22 should no longer be considered high enough to call the pair similar. By calculating a z-score for these specific pairs of proteins, we find for the orthologous alcohol dehydrogenases z(P07327, P00329) = 2.97, and for the unrelated pair of proteins we compute z(Q96LA9, Q8CGP5) = 0.41, now giving a clear distinction on protein functional similarity.

As another example we examine the *PARK2* disease gene, which encodes an E3 ubiquitin protein ligase. Mutations on this gene have been shown to be causative for various forms of Parkinson’s Disease. As a disease gene it is heavily annotated with 91 GO terms in BP ontology, and can therefore be considered an annotation-biased gene that tends to receive on average higher FS scores. In fact, the mean *simGIC/fsBMA* IEA^(+)^ functional similarity score between PARK2 and 1000 randomly selected mouse proteins equals to 0.186, which corresponds to the 99.7 percentile of all human protein scores. In this context, comparison of PARK2 protein (UPANr O60260) with two other mouse proteins, FBXL2 (Q8BH16) and GATAD1 (Q920S3), results in a similar, but very high *simGIC/fsBMA* IEA^(+)^ score of 0.53. However, z(O60260, Q8BH16) = 5.58 whereas z(O60260, Q920S3) = 2.68 provides help to discriminate a protein involved in an E3 ubiquitin-ligase complex (FBXL2) from a zinc finger protein (GATAD1). (All examples can be reproduced on our Frela server).

### Discriminating between Orthologues and Random Protein Pairs

In a recent study, Wu *et al*. used orthology relationships to quantify the ability of a SS measure to distinguish orthologues determined by phylogeny from an equally sized set of randomly paired proteins to demonstrate the superiority of their newly developed SS measure over others^27^. We have extended this idea and made use of high quality orthology relationships to define optimal thresholds for separating pairs of orthologues from random protein pairs for both raw FS scores and z-scores. This allowed us to directly compare FS measures under various conditions, to determine the optimal measure and additionally to investigate possible improvements achieved by applying a z-score calculated on top of the conventional FS scores. Since each measure is evaluated on the same set of cases and controls, all measures considered were equally exposed to any existing annotations bias, shallow annotations, or any other flaws in the GO ontology. It is furthermore known that GO annotations are incomplete and erroneous^44, 45^, and it is not guaranteed that no true positive was selected as a control by random sampling or conversely, that orthologues are not recognized due to very low function similarity described by GO itself. These are challenges to the orthology-based evaluation framework we have applied. However, all measures are tested on the same set of cases and controls, and therefore within this testing framework these effects cancel out when comparing measure performance on a relative basis^9^. Therefore, this allows ranking the tested measures; yet, we discourage to draw conclusions on an absolute scale such as transferring the error rates to other applications.

**Figure 2:**
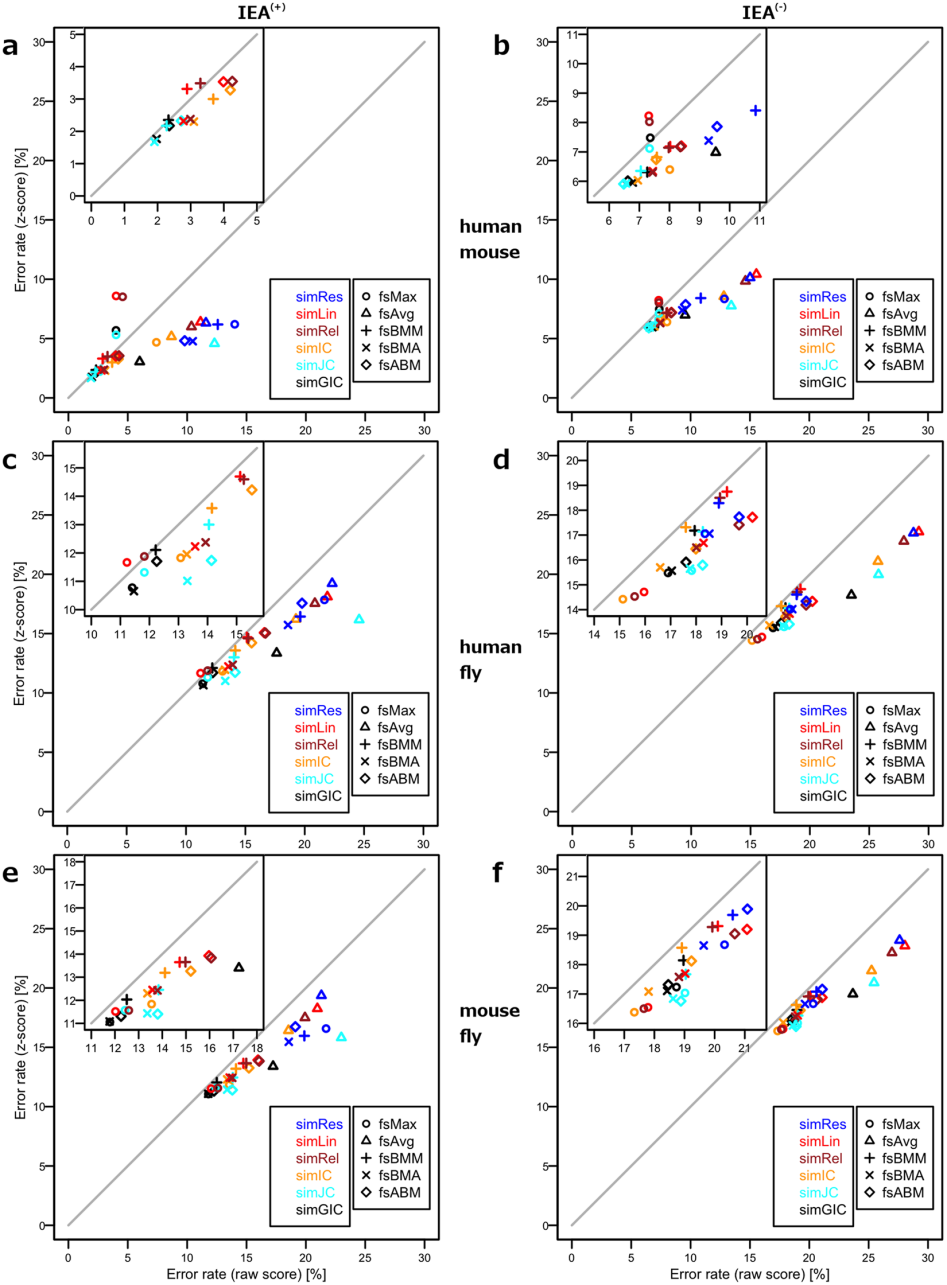
Error rate scatter plots comparing raw and z-scores for different FS scores in BP ontology. Each panel plots the error rates of raw scores (*x*-axis) versus z-scores for pairs of orthologues and controls from selected organisms with a smaller inlay panel in the upper left corner focusing on the best scoring measures. The thick grey diagonal line serves as a measure performance indicator, as any point on this line corresponds to a measure that achieves exactly the same error rate with raw and with z-scores. Any deviation from this line therefore indicates an error rate improvement by either using the raw scores or the z-scores. The first column (panels a,c, and e) presents results from an annotation corpus including electronic annotations, whereas the second column shows the outcome where electronic annotations have been excluded. The rows correspond to the organism pairs used for scoring by orthology: first row, human/mouse; second row, human/fly; third row, mouse/fly. A legend present in each lower right corner gives rise to the colour encoding of SS measures and symbols coding for mixing strategies.

In Fig. 2 we present scatter plots for the percentages of correctly assigned protein pairs after selecting an optimal score threshold for different functional similarity measures using annotations from BP ontology (see paragraph “Benchmarking” in Methods section). These plots therefore show the ideal case where each measure is applying an optimal threshold, which separates best the cases (orthologues) from the controls (random pairs) based on a FS raw score (*x*-axis) and respective z-score (*y*-axis). In general, application of z-scores mildly improved accuracy; FS scores based on *fsMax* are the only exception. The overall lowest error rates were observed for the closely related and well annotated human/mouse orthologues: We found an error rate of approximately 2% (Fig. 2a), when including annotations with IEA evidence codes, and the best measures are *simJC*/*fsBMA* and *simGIC*/*fsBMA*. Interestingly, when excluding IEA evidence codes from the annotation corpus, we saw an increase in error rate to approximately 6%, but the best performing measures were still *simJC* and *simGIC* in combination with either *fsBMA* or *fsABM* (Fig. 2b).

The much more remotely related organisms human and fly posed a greater challenge to functional similarity characterization, reflected by an error rate of about 10.5% for the IEA^(+)^ corpus (Fig. 2c), and *simGIC*/*fsBMA* being again the best performing measure. Here, we found some *fsMax* based scores yielding very low error rates, which became the best performing measure when using IEA^(−)^ data: *simIC*/*fsMax*, *simRel*/*fsMax*, and *simJC*/*fsMax* (Fig. 2d). The good performance can be attributed to the fact that for IEA^(−)^ data *fsMax* behaves similar to the generally well-performing *fsBMA* mixing strategy due to small number of annotations per protein^9^. We have already mentioned the very low error rates for human/mouse orthologues, such that by transitivity the mouse/fly orthologues also yielded similar results as the human/fly comparison. We also found the same measures ranking top. For both IEA^(+)^ and IEA^(−)^, the error rate in mouse/fly orthologues was higher by one to two percent compared to the human/fly orthologue dataset (Fig. 2c,d,e and f).

Notably, introduction of z-scores improved accuracy of the best performing measures only marginally. The biggest gains were observed for *fsAvg*-based measures, which simply compute the average of all entries in the SS matrix. Biologically, *fsAvg* is a rather unsuitable measure to quantify protein functional similarity, as shown by all plots in Fig. 2, where the measure frequently is associated with the highest error rates. By definition, *fsAvg* will perform only well if the two compared proteins are very similar in terms of GO annotations. Since we are comparing orthologues, proteins might already have deviated in function to a certain degree, which can be better picked up by row and column maxima mixing strategies. Remarkably, *fsMax* stood out both positively and negatively. This measure is characterized by the lowest error rate in the human/fly IEA^(−)^ dataset when combined with *simIC*, *simRel*, and *simLin* SS measures (Fig. 2d), but *fsMax*-based z-scores performed worst in combination with *simLin* and *simRel* for human/mouse orthologues when using the IEA^(+)^ dataset (Fig. 2a).

In the MF ontology, z-scores calculated with the *simGIC*/*fsBMA* measure based on the IEA^(+)^ dataset distinguished themselves with the lowest error rates for any set of orthologues we have investigated. When restricting annotations to the IEA^(−)^ dataset, various other measures ranked top, and *simGIC*/*fsBMA* consistently ranked in the top 20% (see Supplementary Fig. S1). This is in line with the results presented by Pesquita *et al*.^9^, who found that both *simGIC* (albeit used as a group-wise measure) and *simRes/fsBMA* performed best in a sequence similarity-based benchmark using MF ontology. Since the CC ontology describes cellular locations, it is not surprising to find rather high error rates when using this ontology for protein functional similarity (see Supplementary Fig. S2). However, as described later in Section “Combining Orthologies”, it increased accuracy when calculating scores from multiple orthologies for a pair of proteins.

In all three ontologies we have observed that *fsBMA* mixing strategy contributed many times to achieve lower error rates. Furthermore, even though several other SS measures were seen in the top ranks, as a general guideline we tend to recommend *simGIC* as the semantic similarity of choice for computing functional similarity. Use of z-scores generally improved measure performance, but especially for *simGIC*/*fsBMA* raw scores and z-scores performed almost equally well. Nevertheless, we see value of z-score usage as an additional discriminatory tool in ranking results, as we have seen in the PARK2 example above. When looking for closely related proteins in a proteome-wide scan, due to its nature *fsAvg* can be a good mixing strategy and based on the results from the orthology relationships, we expect z-scores to be especially beneficial. In most circumstances, *fsMax* should not be used when computing FS scores based on an IEA^(+)^ annotation corpus, as this mixing strategy generally overestimates protein functional similarity. On the other hand, when looking for very good single matches of GO terms, *fsMax* will be a good choice, especially in an annotation corpus without electronic annotations, and has been recommended for protein-protein interaction analysis^32^. Broader partial matches are best detected by *fsBMA*. Overall, we find *simGIC*/*fsBMA* as a recommendable all-round FS measure.

Our results confirm previous findings that inclusion of electronic annotations improves measure accuracy^46, 47^. In particular, Rogers and Ben-Hur^47^ identified a 16% increase in average accuracy as classifier overestimate when predicting protein function using BP ontology and electronic annotations. In BP, when using the IEA^(+)^ dataset we find an average decrease of 3.9% in error rate for both raw and z-score based measures. One reason for this improvement is explained by orthologues that are annotated with only IEA evidence codes such as the pair given by UPANrs (Q9UBX7, Q9QYN3), which is annotated electronically only with GO term GO:0006508 (proteolysis). In our human/mouse orthology dataset, we found 221 orthologous gene pairs where both genes are annotated with IEA evidence codes only, implicating that GO terms have been transferred during an automated annotation process. Moreover, these 221 gene pairs make already 39% of all pairs that are only present in the human/mouse orthologue IEA^(+)^ set, whereas the remaining 61% are pairs where only one protein is annotated exclusively with IEA evidence codes. The improvement with IEA can also be explained by the effect of IEA annotation being partially based on orthology, resulting in a similar GO annotation between orthologues and therefore the similarity measures achieve better discrimination from the background of unrelated proteins. Overall, when including automated annotations, accuracy was higher, but this is also due to minimally and solely electronically annotated proteins, which should be kept in mind when making a decision whether to involve automated annotations. Our web service therefore provides means to investigate FS score composition, so that such cases can be detected easily.

### Choice of Annotation Corpus

The annotation corpus forms the basis for calculation of FS scores and therefore has an influence on the score magnitude, which needs to be kept in mind during score interpretation^43, 46^. It is therefore of interest if protein functional similarity should be computed with data retrieved only from the organism(s) under investigation rather than taking all available data from all annotated organisms. To examine this question within our orthology framework, we restricted the annotation corpus to include only data from the organisms that form the orthologue pairs (AOO) and compared it to our default corpora that do not impose any restrictions on organisms (ALL). For example, for human/mouse orthologues, SS measures were computed from GO graphs containing information content related to only human and mouse protein annotations (AOO), and were compared to SS measures computed from GO annotations of proteins from all organisms (ALL).

When excluding electronic annotations we consistently observed better scoring performance for the BP ontology when using ALL corpus data (see Methods for details on the analysis). On the one hand, this is quite remarkable, as this observation holds for 29 SS/MS combinations, with *simJC*/*fsAvg* being the only exception where the null hypothesis that the two datasets’ mean ranks are equal was not rejected at the significance level of α = 0.01. On the other hand, the magnitude of the difference is rather small: overall, error rate differences are mostly below 1% of the respective case/control dataset size. The measures with the lowest error rates in BP, *simJC* and *simGIC* in combination with *fsBMA* (Fig. 2a), showed only very modest gain of performance when used with ALL corpus data (<0.7% error rate differences). The biggest improvement was found for *simRel*/*fsBMM*, where the (mean) error rate drops by 1.48% from 716.73 (CI = [714.63, 718.83], AOO corpus) to 665.51 (CI = [663.51, 667.50], ALL corpus) false assignments in a dataset of 1726 mouse/fly orthologues and 1726 controls sampled 225 times (Supplementary Fig. S3). A rather similar picture is seen when examining CC ontology, where all SS measures except *simRes* resulted in smaller error rates when computing scores based on ALL corpus data. However, the performance gain is generally larger than in BP ontology, as for example we found a 5.95% drop in error rate for *simIC*/*fsBMM* when using ALL corpus data for human/mouse orthologues (Supplementary Fig. S4). For MF ontology, there were significant improvements for *simLin*, *simRel*, and *simIC* measures with ALL corpus data, but the magnitude in error rate decrease is below 0.5% (Supplementary Fig. S4). Finally, when including electronic annotations there was no clear improvement in using ALL corpus of annotations versus AOO.

To summarize, when discriminating orthologues from unrelated proteins it seems to be beneficial to rely on a larger set of annotation corpora instead of restricting to the annotations in the respective organisms. Even though gain of accuracy is generally small and is only evident when restricting to IEA^(−)^ annotations, it is much more convenient to compute scores based on a single database derived from all available annotation corpora instead of using corpora tailored to organisms.

### Combining Ontologies

A decade ago, Schlicker *et al*. suggested combining FS scores from BP and MF ontologies into a single FS score by computing the root mean square of the two ontologies^48^, which to our knowledge has never undergone any performance investigations. The orthology testing framework described here, provided an appropriate environment for investigating the performance of FS measures based on BP+MF combined ontologies, as well as the effect of combing all three ontologies (BP+MF+CC) into a single FS score. Our analysis of BP+MF, and BP+MF+CC combined ontology FS scores reflects an increasingly specific filtering strategy starting with biological processes: an annotation to BP is made when a gene product activity is part of, or regulates, or is upstream of but still necessary for, a biological program^49^. This broad context makes BP annotations especially valuable for protein functional similarity calculations. Requiring additional similarity by MF, that is, comparable biochemical or signalling activity, will generally narrow down the list of matching candidate proteins and increase the overall sensitivity of the query. Demanding protein colocation by utilizing annotations from CC ontology presents the ultimate refinement in protein functional similarity that can be retrieved from GO. For completeness, the Frela server allows score calculation based on any combination of ontologies.

When looking at the results we need to keep in mind that combined ontologies require every protein be annotated to each single ontology, which results in a smaller number of orthologous gene pairs used in the assessment (see Table 2). For example, there are 2240 human/fly orthologues annotated in BP, including automated annotations. However, there are only 1868 orthologues annotated to both BP and MF ontologies, and this number drops to 1516 for orthologous gene pairs with annotations in all the ontologies.

**Table 2:** Number of orthologous gene pairs used in this study.

Ontology	Human/fly^a^	Human/mouse^a^	Mouse/fly^a^
BP	2240 (1851)	2361 (1798)	2171 (1726)
MF	2081 (1679)	2189 (1627)	1999 (1530)
CC	1958 (1710)	2719 (2035)	1946 (1694)
BP+MF	1868 (1405)	1996 (1422)	1763 (1258)
BP+MF+CC	1516 (1199)	1904 (1283)	1422 (1052)

^a^Numbers refer to IEA^(+)^ data sets, whereas numbers in parentheses refer to IEA^(−)^ data sets.

**Figure 3:**
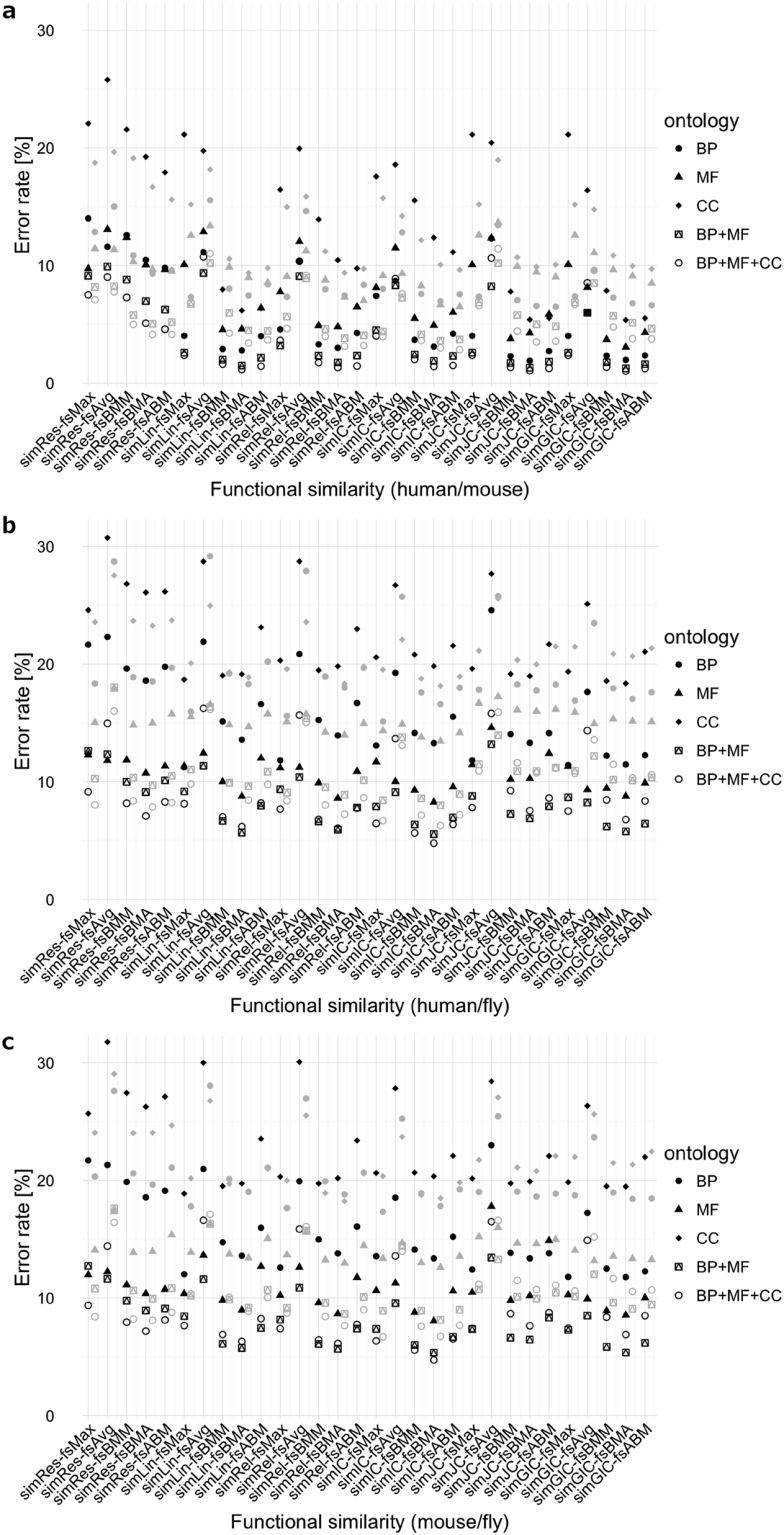
Error rates of various FS measures computed for different ontologies. Each panel shows error rates for different measures (*x*-axis, measure names separated by a hyphen) for all three gene ontologies and the two combined ontologies (encoded by symbols, see legend to the right of each panel). Error rates for IEA^(+)^ dataset-derived scores are shown in black, and the corresponding scores calculated for the IEA^(−)^ dataset are depicted as grey symbols in a separate column to the right of the corresponding IEA^(+)^ results. Since the error rates are given as percentages, we refer to Table 2 for dataset sizes and Supplementary Data File S1 for underlying raw data. Panels represent all orthology relationships that entered this study: (**a**) human/mouse orthologues; (**b**) human/fly orthologues; and (**c**) mouse/fly orthologues.

In general, we observed substantially lower error rates in all three orthology data sets in comparison to the scores based on a single ontology (Fig. 3). Even though FS measures involving the CC ontology showed the highest error rates of all three ontologies, inclusion of this ontology further improved the error rates when compared to BP+MF combined ontologies, especially for the closely related human/mouse organisms (Fig. 3a). Also, use of combined ontologies tends to level out the performance of FS measures, in the sense that error rate differences became smaller.

We observed that for the majority of measures applied to human/mouse orthologues, the BP+MF+CC combined ontology is superior to any other single or combined ontology within the IEA^(−)^ dataset, with *fsAvg* and *simLin*/*fsMax* being the only exceptions (Fig. 3a, grey circles).

**Figure 4:**
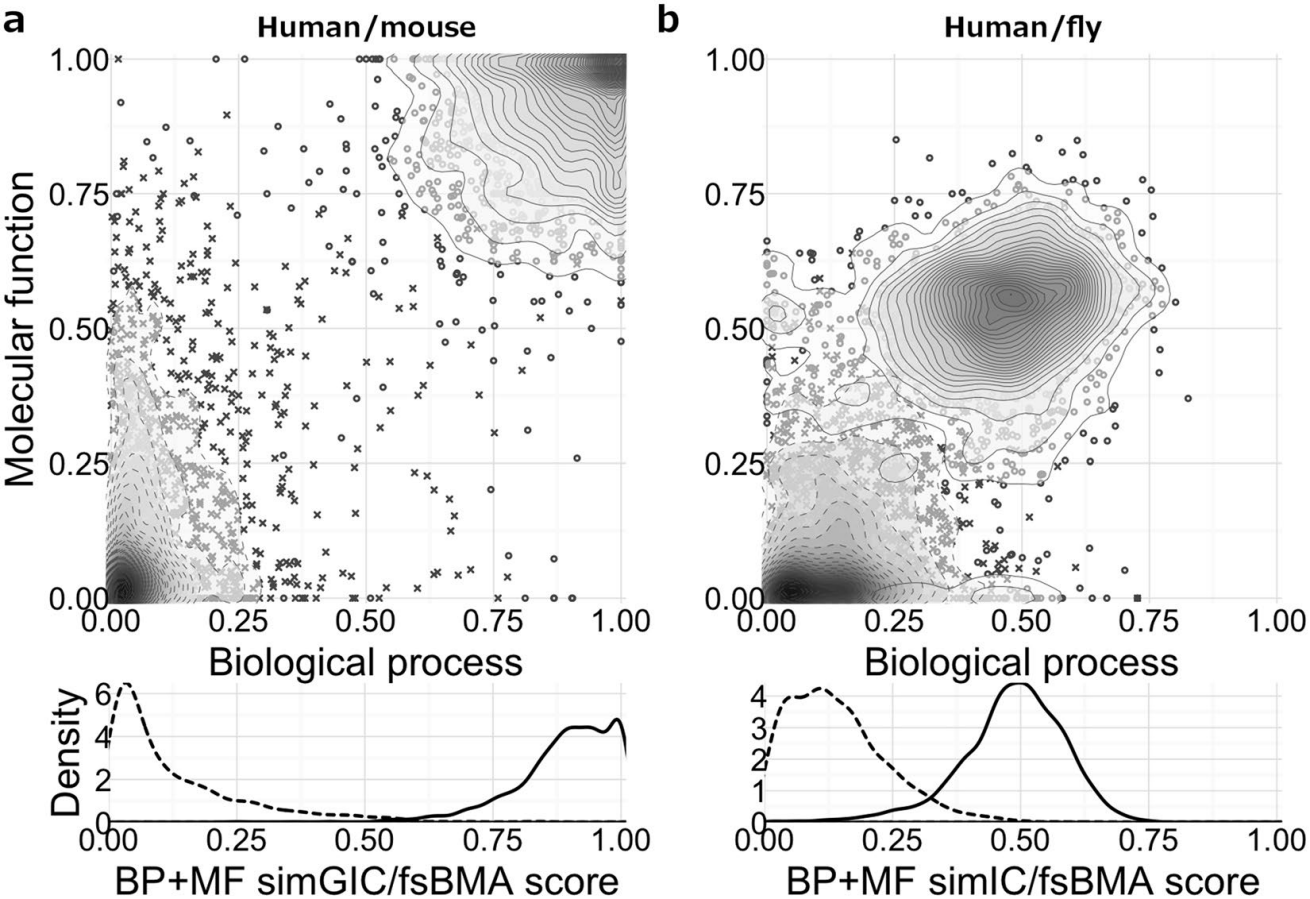
Distribution of BP, MF, and BP+MF combined FS scores (IEA^(+)^ dataset) for selected orthologies. The upper part of the figure shows a scatter plot of BP (*x*-axis) and MF (*y*-axis) scores of orthologous gene pairs (cases, displayed as circles) and randomly selected gene pairs (controls, visualized as crosses) from the respective organisms. On top of this scatter plot the two-dimensional density function of these two distributions is displayed by solid and dashed iso-lines for cases and controls, respectively. Each two-dimensional point in this scatter plot is mapped to a real value by the *F*_BP+MF_ function, which is the root mean square of the two individual scores. In the bottom part of the figure we show the one-dimensional density function of the so-computed *F*_BP+MF_ scores for cases and controls using the same line styles as above. The crossing point of the two density graphs defines the optimal threshold for minimizing the error rate. (**a**) Human/mouse orthologues and controls with their associated *simGIC*/*fsBMA* scores. The *simGIC* semantic similarity in conjunction with the *F*_BP+MF_ function greatly separates cases from controls, with an error rate of only 1.24%. (**b**) Human/fly orthologues and controls with their associated *simIC*/*fsBMA* scores. On average, we find 207.28 (CI = [205.86, 208.69]) incorrectly assigned pairs out of 1868 cases and 1868 controls, which corresponds to an error rate of 5.55%.

In Fig. 4 we highlight two measures that, when using electronic annotations, ranked top in the BP+MF human/mouse and human/fly combined ontology, respectively (also see Fig. 3a and b). Utilizing *simGIC*/*fsBMA*, orthologues from the closely related human and mouse organisms are very well separated from their controls, for both BP and MF ontologies (Fig. 4a, upper part). The corresponding two peaks in the combined BP+MF score are accordingly well separated (Fig. 4a, lower part), such that the overall error rate is only 1.24%. For the more remotely related organism pair human/fly, the densities for cases and controls calculated with the *simIC*/*fsBMA* measures overlap by some extent (Fig. 4b). Notably, there is a smaller fraction of orthologues that do not share any similarity in the MF ontology, but do have considerable high BP scores (Fig. 4b, circles on the upper half of the *x*-axis). One of these orthologues is given by the human/fly pair Q6ZYL4/B7Z018 (raw score in BP is 0.50, z-score is 5.09), but due to only a single annotation in MF for each of the two proteins, which evaluates to a raw MF score of 0.0, and therefore heavily penalizes calculation of the combined score, this orthologous pair became a false negative in the BP+MF combined ontology.

The results indicate that there are considerable differences between established FS scores, where some measures perform better in the identification of functionally related proteins. In addition, the results suggest that combining FS scores from the three ontologies (BP, MF, and CC) tends to improve the performance of the various FS measures. Future work should address several remaining issues, in particular problematic cases should be investigated, for example where functional similarity scores are unexpectedly low, in order to identify the limitations in the current functional similarity approaches and devise new solutions. Many approaches have been proposed for measuring functional relationships, but in the current work we have only assessed a few of the most established. It would be desirable to extend the current analysis to other approaches, as for example group-wise SS measures. Finally, the current work focused on orthologous genes as a reference set of functionally related proteins, future work should focus on investigating FS-based approaches for concrete applications like candidate gene prioritization.

### Web Server

**Figure 5:**
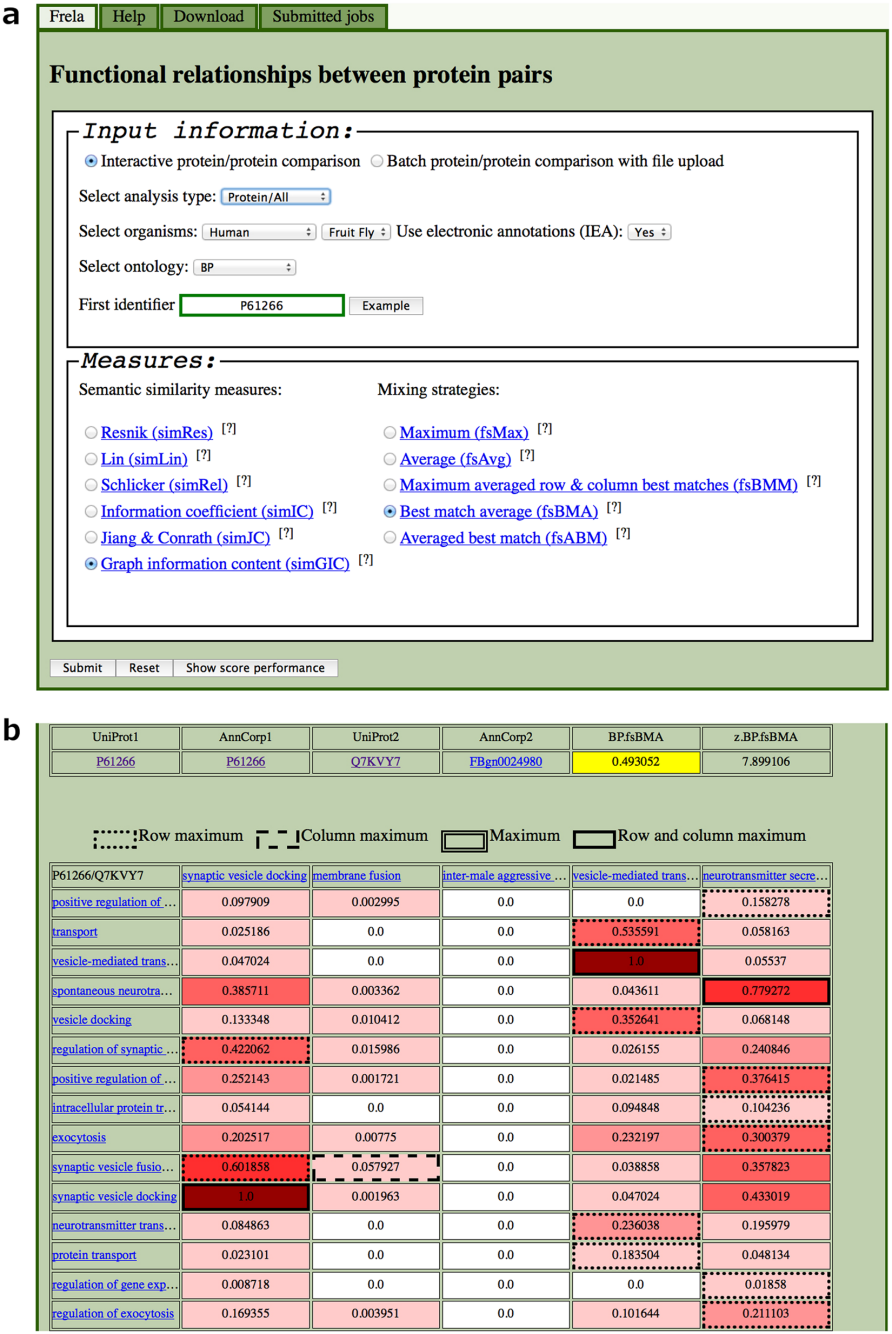
Screenshot of Frela web interface. (**a**) Main input web form. This form provides all necessary elements to run a protein functional similarity calculation with Frela. The user can choose between manual data entry (interactive protein-protein comparison) and file upload (batch mode). We support computation of pairwise protein functional similarities for any organism or a scan of a single protein versus all annotated human, fly, or mouse proteins. In addition, it is possible to specify use of either the IEA^(+)^ or the IEA^(−)^ dataset with the “Use electronic annotations (IEA)” option. In this panel we show the parameters used in the example given in the “Web Server” section, which is a scan of the human STX1B protein (UPANr P61266) versus all annotated proteins from the fly BP ontology, calculating *simGIC*/*fsBMA* scores and including IEA evidence codes. (**b**) Semantic similarity score matrix for the pair of syntaxin proteins given by UPANrs P61266 and Q7KVY7 with the same parameters as in panel (a). Since the FS score is computed from pairwise SS scores derived from the GO annotations from the respective proteins being compared, this SS score matrix provides important insight into FS score calculation and interpretation. The background of a cell is a colour gradient, which corresponds to the SS score and ranges from white (no similarity) to dark red (highest similarity). Cells involved in FS score composition are surrounded by thick black lines, and the line style informs about the type of maximum the cell contains: row maximum, dotted line; column maximum, dashed line; row and column maximum, solid line. The *fsBMA* score is then calculated from the sum of row maxima (6.279957) and column maxima (2.837199) as 1/2× (6.279957/15 + 2.837199/5) = 0.4930518, since in BP, P61266 is annotated with 15 GO terms (number of rows) and Q7KVY7 is annotated with 5 terms (number of columns). The third column of this SS matrix corresponds to GO term GO:0002121, “inter-male aggressive behavior”, which does not share any similarity with any of the GO terms from the human protein. Dropping this term from the annotation of the fly protein results in an improved *fsBMA* score of 0.563981775.

Our work is summarized in the Frela web server located at http://frela.eurac.edu, which supports calculation of different FS measures by combining any of the six SS measures with five different mixing strategies discussed in this study. Frela can be invoked in two major modes: the interactive mode receives input directly from the user through web entry forms, while the batch mode processes uploaded files. It is possible to perform calculations on either the IEA^(+)^ or the IEA^(−)^ dataset for any of the BP, MF, and CC ontologies or combinations thereof (Fig. 5a). For similarity computations involving proteins from human, mouse, or fruit fly organisms, the server accepts UniProt/SwissProt accession numbers as protein identifiers. Since internally we are using the MySQL database provided by the GO consortium, which uses gene or protein identifiers as supplied by contributors, other organisms’ identifiers must match those stored in the database. For example, the Arabidopsis thaliana *HSK* gene, which codes for a homoserine kinase, is deposited under TAIR accession code “locus:2827533” in the GO database and as such can be used as query input to Frela. For further information, we refer to the GO web page, which offers all necessary details on stored gene and protein identifier systems.

Both the interactive and batch mode allow input of protein pairs as input. Any identifier entered in the interactive mode will activate the organism auto detection and conveniently preselect an organism name in the corresponding dropdown menu. In case no matching UniProt protein accession code has been found in the server’s list of annotated human, mouse or fly accession codes, we assume a different organism with the appropriate identifier has been entered. At this point it is important to underline that due to expensive precalculations, the web server exclusively computes protein FS z-scores for comparisons between human, mouse, and fly organisms with protein identifiers provided as UniProt accession codes.

In interactive mode, we furthermore support scanning a protein versus all annotated proteins for a chosen ontology. Execution time depends on the chosen parameters and especially on the number of annotations made to the query protein. For example, a *simRel*/*fsBMA* run of a human protein annotated with 15 GO terms in BP versus all BP-annotated fly proteins including IEA evidence codes requires roughly 9000 pairwise score calculations, which is completed after 15 seconds.

In addition to score computation of protein pairs, batch mode conveniently offers all versus all score calculation of two uploaded files containing protein identifiers. This is especially useful when scoring a list of proteins versus a panel of proteins of interest, such as a disease-related set of proteins.

After calculations are finished, a downloadable result table is displayed, which is sorted by z-score or raw score, depending on the input parameters. For each row in this table, the semantic similarity matrix can be displayed by clicking on the corresponding score. The matrix is composed of GO term names linked to the GO website and color-codes term SS scores from white (no similarity) to red (high similarity). Based on the chosen FS measure, various types of maxima are denoted by line styles of the affected cells, which greatly simplify understanding score calculation and further score interpretation.

As an example we have selected the human syntaxin gene *STX1B* (UPANr P61266), which has been associated with Parkinson’s Disease^50^. When evaluating *simGIC*/*fsBMA* scores for the BP IEA^(+)^ ontology, its fly orthologue *Syx1A* (UPANr Q7KVY7) ranks on position 50 of the hit list sorted by z-score (and position 107 when sorted by raw score). One reason for this rather low rank is that the fly protein is annotated with GO term GO:0002121, “inter-male aggressive behavior”, a term which is obviously missing in the annotation of the human protein (Fig. 5b). This results in a lower *fsBMA* score and therefore worsens its rank in the similarity hit list. On the other hand, this pair receives the highest z-score when running the scan for the MF ontology. Most importantly, calculating combined BP+MF scores ranks the pair of orthologues on top position, underlining the power of combining scores from different ontologies.

The abovementioned examples can easily be reproduced using the web server located at http://frela.eurac.edu, where each calculation takes less than a minute. Furthermore, the web server provides interactive versions of the graphs presented in Fig. 2, facilitating choice of an appropriate functional similarity score. The web server is freely available, and the source code can be downloaded from our web site.

## Methods

An annotation corpus *A* establishes a relationship between a GO term *t* and gene *G* (or gene product). We have retrieved GO graphs and annotations as of September 2015 as MySQL dumps from the GO website and extracted data for human and the model organisms Drosophila melanogaster (fly) and Mus musculus (mouse), which form the basis for all of our comparisons. For each of the BP, MF, and CC ontologies we compute the term probabilities *P*(*t*) = *N*(*t*)*/N*(*root*), where *N*(*t*) denotes the number of proteins annotated with a term *t* or any of its descendants, and *root* is the ontology’s unique term without any ancestors. The GO term information content is then given by *I*(*t*) = −log(*P*(*t*)).

### Semantic and Functional Similarity Measures

In this study we concentrate on six frequently used pairwise SS measures. Let therefore *s* and *t* be two GO terms we want to compare semantically and let *S*(*s*, *t*) denote the set of all common ancestors of *s* and *t*. The measure defined by Resnik^51^ is given by

1 $${simRes}(s,t)=\,\mathop{{\rm{\max }}}\limits_{c\in S(s,t)}I(c),$$

whereas Lin’s measure^52^ takes into account the information content of the most informative common ancestor relative to the information content of the two terms,

2 $${simLin}(s,t)=\,\mathop{{\rm{\max }}}\limits_{c\in S(s,t)}\frac{2\cdot I(c)}{I(s)+I(t)}.$$

Schlicker weights Lin’s measure by common ancestor term probability^39^,

3 $$simRel(s,t)=\,\mathop{max}\limits_{c\in S(s,t)}(\frac{2\cdot I(c)}{I(s)+I(t)}\cdot (1-P(c))),$$

and a similar approach has been proposed independently^53^ by introducing the information coefficient measure as

4 $${simIC}(s,t)=\,\frac{2\cdot \mathop{\max }\limits_{c\in S(s,t)}I(c)}{I(s)+I(t)}\cdot (1-\frac{1}{1-\mathop{{\rm{\max }}}\limits_{c\in S(s,t)}I(c)}).$$

Jiang and Conrath’s measure^33^ has been shown to be equivalent with Lin’s measure^35^ but is included in our work for historical purposes. It is defined by

5 $${simJC}(s,t)=\frac{1}{1+I(s)+I(t)-2\cdot \mathop{{\rm{\max }}}\limits_{c\in S(s,t)}I(c)}.$$

Finally, we use the graph information content measure^9^, which by design is a group-wise SS measure, as a pairwise measure for consistency as suggested by Li *et al*.^53^:

6 $${simGIC}(s,t)=\frac{{\sum }_{c\in \{S(s,s)\cap S(t,t)\}}I(c)}{{\sum }_{c\in \{S(s,s)\cup S(t,t)\}}I(c)}$$

Let us assume protein *P* is annotated with *m* GO terms *t*_*1*_, *t*_*2*_,.., *t*_*m*_ and protein *R* is annotated with *n* GO terms *r*_*1*_, *r*_*2*_,.., *r*_*n*_, then the matrix *M* is given by all possible pairwise SS values *s*_*ij*_ = *sim*(*t*_*i*_, *r*_*j*_) with *sim* being one of the SS measures introduced above, *i* = 1, 2,.., *m* and *j* = 1, 2,.., *n*. Functional similarity is computed from the SS entries of *M* according to a specific mixing strategy (MS), and in this work we investigate five different mixing strategies. A frequently used MS uses the maximum value of the matrix, ${fsMax}={{\rm{\max }}}_{i,j}{s}_{ij}$, whereas *fsAvg* takes the average over all entries, ${fsAvg}=\frac{1}{m\times n}\sum _{i,j}{s}_{ij}$. Furthermore, using the maximum of averaged row and column best matches has been suggested for incomplete annotations, ${fsBMM}=\,{\rm{\max }}(\frac{1}{m}\sum _{i}{{\rm{\max }}}_{j}{s}_{ij},\frac{1}{n}\sum _{j}{{\rm{\max }}}_{i}{s}_{ij})$. Instead of taking the maximum, averaging gives the so-called best match average ${fsBMA}=\frac{1}{2}(\frac{1}{m}\sum _{i}{{\rm{\max }}}_{j}{s}_{ij}+\frac{1}{n}\sum _{j}{{\rm{\max }}}_{i}{s}_{ij})$, and conversely, the averaged best match is defined as ${fsABM}=\frac{1}{m+n}(\sum _{i}{{\rm{\max }}}_{j}{s}_{ij}+\sum _{j}{{\rm{\max }}}_{i}{s}_{ij})$.

We additionally study the effect of combining multiple gene ontologies into a single score, as suggested previously^48^. We focus on pooling scores from BP and MF ontologies and from BP, MF, and CC ontologies. A functional similarity *F* is computed by combining a SS measure with any mixing strategy defined above over any of the different ontologies: biological process (*F*_BP_), molecular function (*F*_MF_), and cellular component (*F*_CC_). We compute the combined measures as the functions ${{F}}_{BP+MF}=\sqrt{\frac{1}{2}({{F}}_{BP}^{2}+{{F}}_{MF}^{2})}$ and ${{F}}_{BP+MF+CC}=\sqrt{\frac{1}{3}({{F}}_{BP}^{2}+{{F}}_{MF}^{2}+{{F}}_{CC}^{2})}$.

### Z-score calculation

Given a pair of target organisms, for each annotated protein in the respective corpora we compute a score background distribution by random sampling without replacement of 1,000 annotated proteins from the corresponding second organism. From this distribution we compute the mean and standard deviation. For example, if we choose human and mouse, in our dataset we have 21,212 human proteins furnished with BP annotations (excluding IEA) and therefore yield 21,212 mean values and standard deviations, one pair for each annotated protein. Conversely, we find 9,714 manually annotated proteins for mouse (see Table 1). Guided by the Central Limit Theorem for the sum of independent random variables, we define for a pair of proteins *P* and *R* the similarity z-score as

7 $${z}({P},{R})=\frac{2\times {F}({P},{R})-({{\mu }}_{{P}}+{{\mu }}_{{R}})}{{({{\sigma }}_{{P}}^{2}+{{\sigma }}_{{R}}^{2})}^{1/2}},$$

where μ and σ denote the mean values and standard deviations for proteins *P* and *R*, respectively, and *F*(*P*, *R*) is a functional similarity measure between *P* and *R* based on a combination of any supported SS and MS. From the definitions above it follows that the mean values and standard deviations depend on the ontology, the organism, and the functional similarity, but for reasons of simplicity these parameters are omitted in the formula.

An alternative approach to score the protein pair (*P*, *R*) is provided by a modified z-score^54^, which utilizes medians instead of means and may be considered for distributions deviating heavily from Gaussians. We have reviewed its applicability, and for most of our functional similarity measures we find only small differences between means and medians in representative sets of randomly paired proteins. We therefore remain with the more traditional definition of z-scores described above. Further discussion concerning modified z-scores is provided in Section “Modified z-Score” in the Supplementary Material.

### Annotation Corpora

We investigate the influence of various compositions of annotation corpora on FS scores (hereafter briefly called *scores* or *raw scores* when a distinction to z-scores is needed). First, since there is much discussion about the impact of automated annotations, we utilize datasets that include data inferred from electronic annotations (IEA^(+)^) and such that do not (IEA^(−)^). The latter is implemented by omitting annotations to GO evidence code IEA. Simultaneously, we examine the effects of incorporating annotation data from organisms other than those being compared. Whenever we compare proteins from our set of target organisms (human, fly, mouse), we distinguish between scores calculated with all available annotations in GO (ALL corpus) and scores that are based only on annotations made to the organisms belonging to the proteins under investigation (AOO corpus).

GO delivers annotations to genes and gene products, however, organisms are annotated with different gene identifier systems. In our case, mouse and fly gene identifiers are internally mapped to UniProt accession codes through the respective mappings retrieved from Ensembl 82 Biomart, which has been released in September 2015, matching the release date of the GO database we use. Human annotations do not undergo identifier mapping, as GO already delivers annotations using UniProt accession codes.

### Benchmarking

We use orthology relationships as a means to evaluate a measure’s ability to discriminate between functionally related and unrelated pairs of proteins: orthologous genes should have similar functions and therefore we expect them to have higher scores than randomly selected pairs of genes. In particular, we use Ensembl 82 Biomart to retrieve one to one orthology relationships between human/mouse, human/fly, and mouse/fly organism pairs of protein coding genes^55^. In order to remove trivial cases, we exclude orthologous pairs with 80% sequence identity or higher (see Table 2). The list of orthologous pairs serves as cases and an equally sized matched control set is constructed by randomly replacing the orthologous gene with another gene from the same organism (without replacement and without fixed points). Given a score threshold *h*, we assign true and false positives (*TP* and *FP*), and true and false negatives (*TN* and *FN*) according to standard nomenclature^56^. We then determine the optimal threshold *h* ^*^, for which the error rate is minimized. The error rate (or fraction of incorrect) is the number of incorrectly assigned pairs divided by the number of all pairs, (*FP* + *FN*)/(*TP* + *FP* + *TN* + *FN*). Since this error rate calculation involves randomly drawn controls, the outcome will differ from one random control set to another. We therefore repeat optimal threshold determination and its associated error rate computation on 225 sets of control proteins and compute 99% confidence intervals (CI) of the mean for the so-obtained 225 error rates of both raw scores and z-scores. In this manuscript, we therefore always report mean error rates of 225 individual error rates, called “error rate” for brevity. Accordingly, reported thresholds, especially those shown in the Frela web interface, refer to an average over 225 optimal thresholds *h* ^*^. We refer to Supplementary Data File S1 and Supplementary Fig. S7 for a comprehensive list of optimal thresholds, error rates, and confidence intervals for all three orthology pair sets, ontologies and FS measures.

We specifically test if there are SS/MS measures that significantly perform better (or worse) when using the AOO annotation corpus instead of the ALL corpus. For an arbitrary fixed ontology and a SS/MS measure, we perform a Wilcoxon signed-rank test that compares the individual error rates based on z-scores of each of the 225 case/random control datasets between ALL and AOO corpora (with IEA evidence code annotations), using cases from all three orthology relationships we investigate, human/mouse, human/fly, and mouse/fly. In order to detect whether the ALL or the AOO corpus results in better error rates, we perform one-sided tests. A test is considered significant at the level of α = 0.01 after Bonferroni adjustment for multiple hypothesis testing of 30 measures (six SS measures times five mixing strategies).

### Implementation

Our software extends the Dintor framework^57^ for functional similarity analysis and is implemented in the Python programming language. We make use of a client/server architecture, where the computation server is decoupled from the web server. This specifically allows employing a locally installed server that is queried by users from different hosts with computationally inexpensive client software. On a 2.3 GHz AMD Opteron processor with 32GB of RAM, Frela computes BP protein functional similarity scores of 10,000 random protein pairs from the human organism in about 10 seconds and scales linearly with the number of protein pairs compared. Time doubles when utilizing the *simGIC* SS measure due to more complex graph algorithms used in score calculation (for a more complete set of timings, see Supplementary Table S1). The web front end is served through Apache and supports various calculation modes with an emphasis on visualization of functional similarity score derivation. The package can be downloaded from our web server, http://frela.eurac.edu.

## Electronic supplementary material

Supplementary Material

Data File S1

Data File S2
