# Supplementary material for: Exploring Approaches for Detecting Protein Functional Similarity within an Orthology-based Framework
Source: Sci Rep. 2017 Mar 23;7:381. doi: 10.1038/s41598-017-00465-5 (PMC5428484; doi:10.1038/s41598-017-00465-5)
Supplement: Supplementary file 1 — Supplementary Material [file 41598_2017_465_MOESM1_ESM.pdf]

Supplementary Information: Exploring Approaches for Detecting Protein Functional Similarity within an Orthology-based Framework

Christian X. Weichenberger[1,*], Antonia Palermo[1], Peter P. Pramstaller[1], and Francisco S. Domingues[1]

[1]Center for Biomedicine, European Academy of Bozen/Bolzano (EURAC), Viale Druso 1, 39100 Bolzano, Italy.

[*] Corresponding author, email: christian.weichenberger@eurac.edu

Supplementary Information

In this text, we present Supplementary Figures S1 and S2, which are the equivalent plots to Figure 2 in the main text, generated for MF and CC ontology. Supplementary Figures S3 and S4 represent comparisons of error rates between ALL and AOO-based corpora for BP, MF, and CC ontologies. A comparison between z-scores is performed in Section “Modified z-Score”, with results summarized in Figures S5 and S6. The confidence intervals of the error rates are visualized in Figure S7. Supplementary Table S1 summarizes runtime measurements for various Frela invocations.

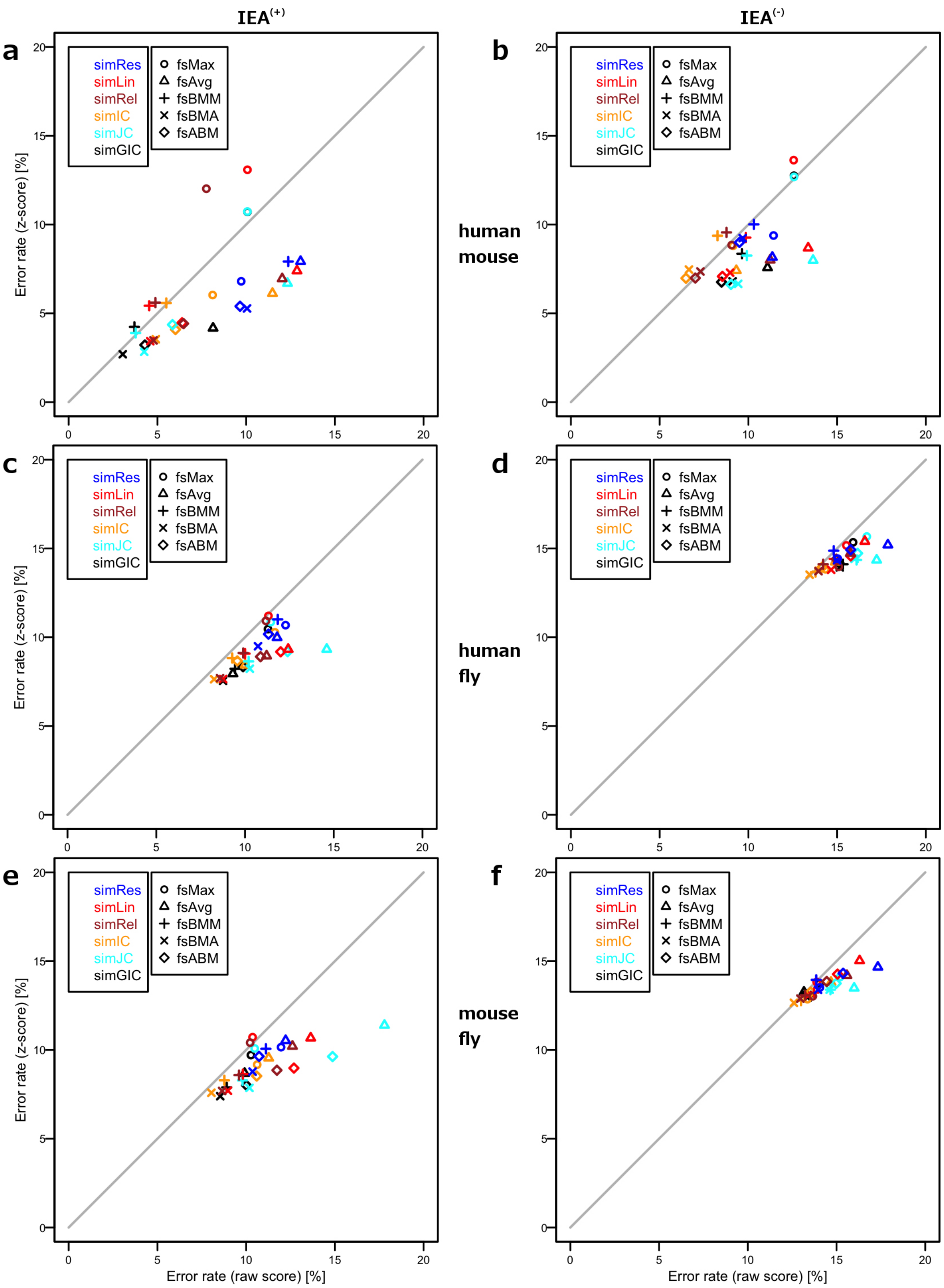

Supplementary Figure S1. Error rate scatter plots comparing raw and z-scores for different FS scores based on MF ontology. See main manuscript Figure 2 for a detailed description on the organization of this figure. Error rates show the same characteristics as in the BP ontology. We frequently find *fsBMA* and to a lesser extent *fsABM* mixing strategies among the best scoring FS measures. Measure *simGIC/fsBMA* again shows very good overall performance.

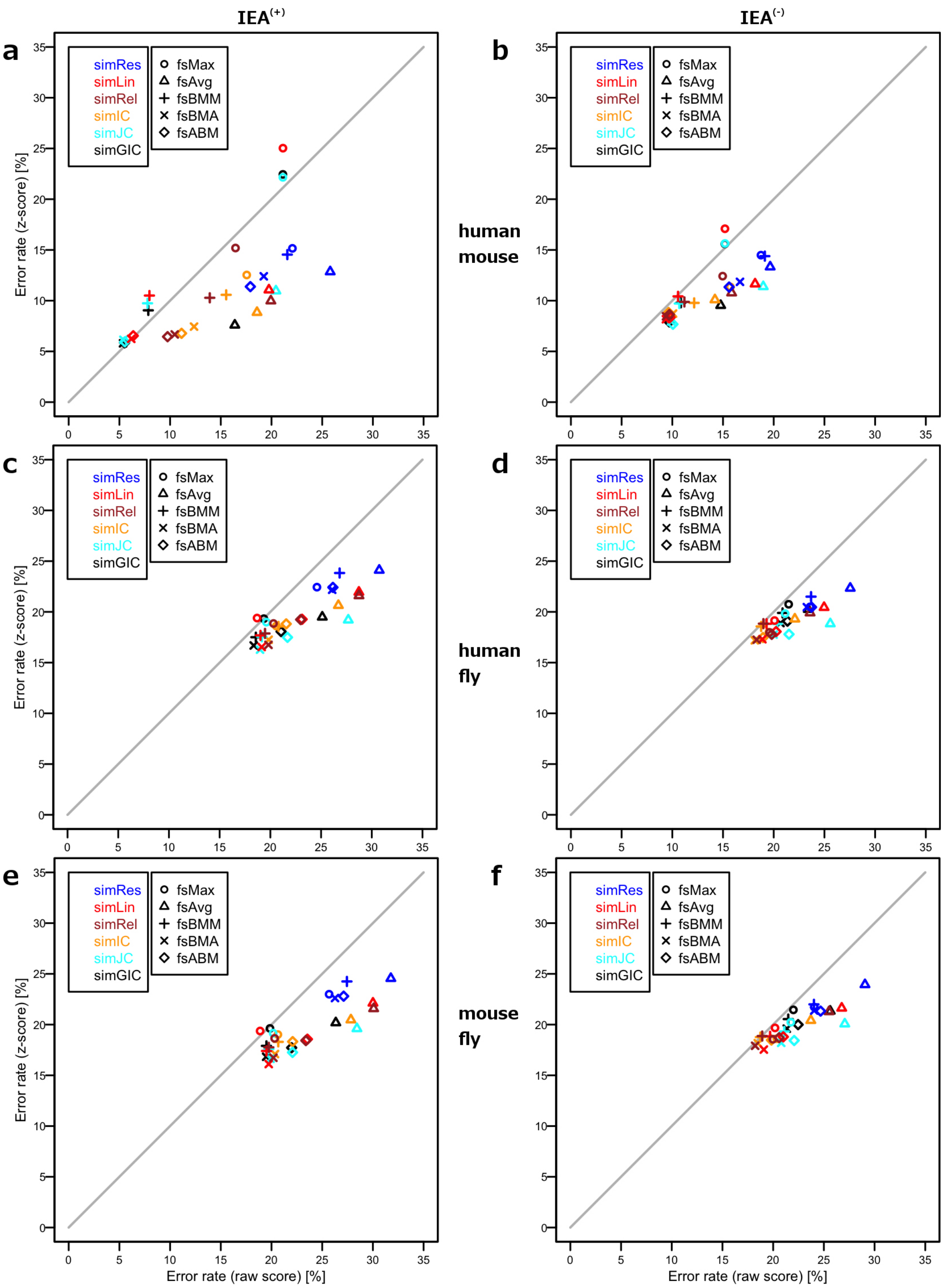

Supplementary Figure S2. Error rate scatter plots comparing raw and z-scores for different FS scores based in CC ontology. See main manuscript Figure 2 for a detailed description on the organization of this figure. Using cellular location to predict protein functional similarity generally results in higher error rates than in BP and MF ontologies, suggesting that location information less accurately describes protein function. Nevertheless, error rates are mostly below 20%, therefore location data still contain valuable information, which improve accuracy of combined ontology BP+MF+CC.

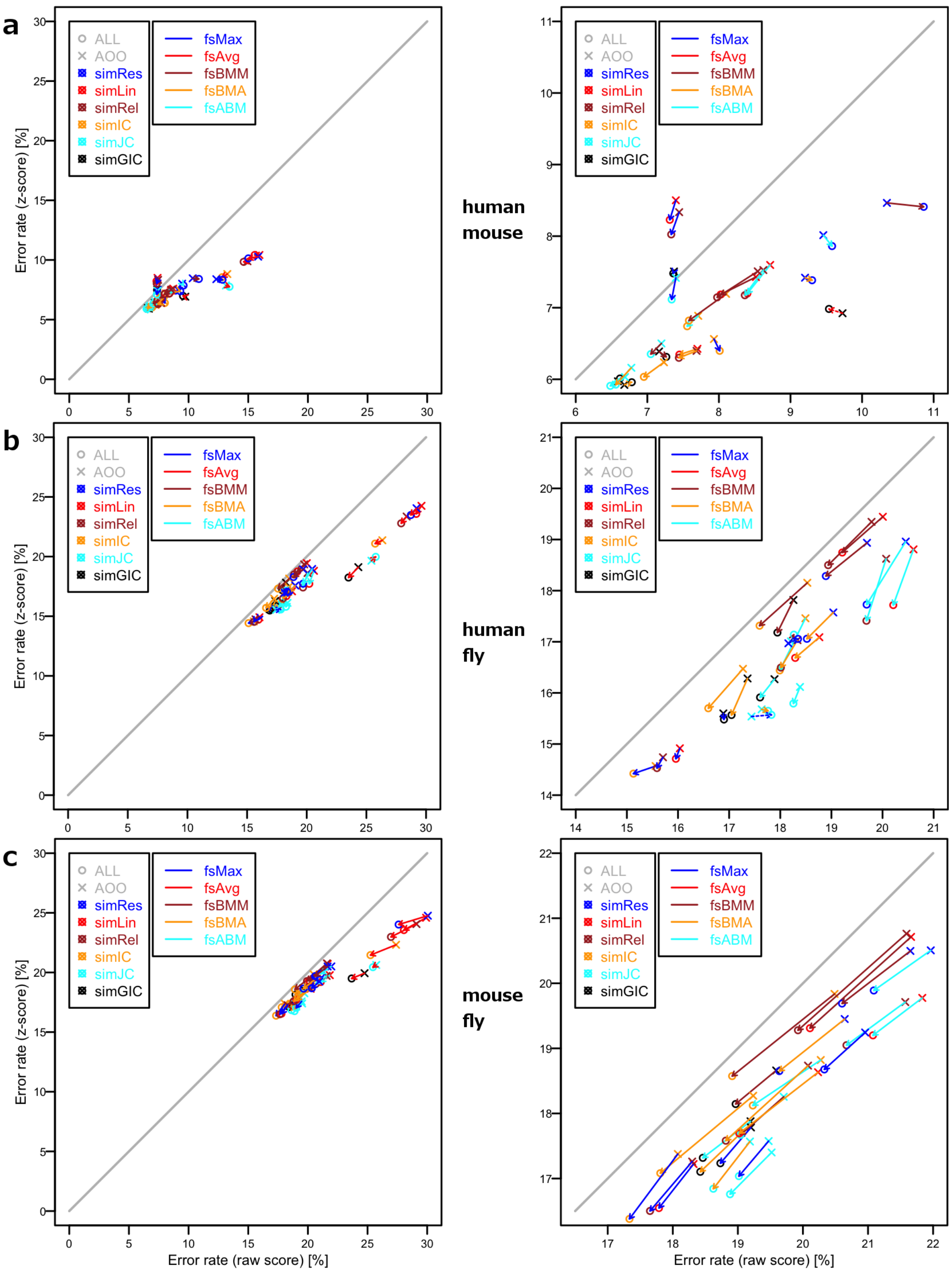

Supplementary Figure S3. Comparison of error rates obtained by using ALL- and AOO-based corpora for BP ontology. Error rates for the respective orthologue pairs are scatter-plotted on the same scale (left column) and magnified (right column, varying scale and origin), with raw scores shown on the x-axis and z-scores on the y-axis. We encode ALL/AOO error rates for SS/MS combinations by coloured crosses (AOO error rate) and circles (ALL error rate) connected by a coloured arrow (mixing strategy). This arrow is pointing toward the ALL error rate and is represented by a solid line if the z-score error rate decreases when using ALL corpus data instead of AOO, and is dashed otherwise. Almost all SS/MS measures exhibit decreased z-score error rates when utilizing ALL corpus data.

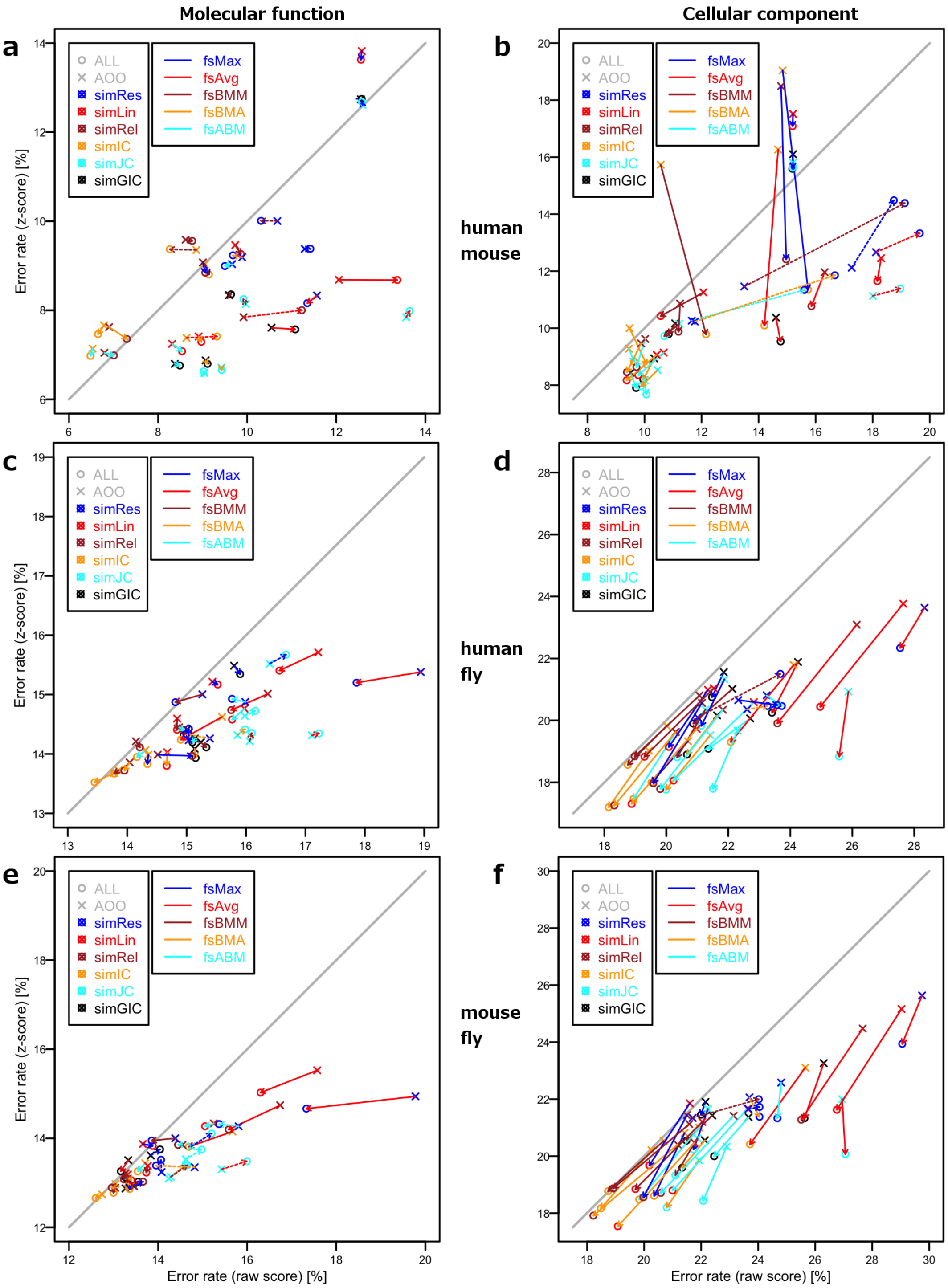

Supplementary Figure S4. Comparison of error rates obtained by using ALL- and AOO-based corpora for MF and CC ontology. Plots follow the same style as Supplementary Fig. S3, but show magnifications only and are therefore on varying scale and with different origin. Whereas only a fraction of SS/MS measures improve by using ALL corpus annotations in MF ontology (left column), the majority of measures has lower z-score error rates when scoring with an ALL corpus in CC ontology(right column).

Modified z-Score

We use z-scores to transform functional similarity measures to units that are centered about the mean, and which account for the spread of the underlying distribution. When this distribution is Gaussian, z-scores can be interpreted quantitatively, e.g. an observed value x with z-score of +2 informs that 97.7% of all observations in the distribution are less than this particular value. A z-score is computed as $z(x) = (x - \mu)/\sigma$, where μ and σ are the mean and the standard deviation of the distribution, respectively. When distributions are known to be non-normal, an alternative is given by the modified (also known as robust, or non-parametric) z-score $z'(x) = (x - m)/d$, where m is the median of the distribution and d is the median absolute deviation, $d = \text{median}(|x_i - m|)$. In order to see if use of a modified z-score would have a strong impact on the benchmark results, we investigate the differences between mean and median, $\mu - m$, and standard deviation and mean absolute deviation, $\sigma - d$.

We have scored 50000 random protein pairs for each of the organism pairs human/mouse, human/fly, and mouse/fly in the BP ontology excluding IEA annotations. This resulted in 3 × 6 × 5 = 90 [organism pairs × semantic similarity measures × mixing strategies] score distributions, for which we then have computed the mean, median, standard deviation, and median absolute deviation, with the R functions `mean`, `median`, `sd`, and `mad`, the latter used as a consistent estimator for the estimate of the standard deviation. (These values are provided in Supplementary Data File S2.) Supplementary Figure S5 shows a scatter plot of the percentage differences between mean and median, and standard deviation and median absolute deviation, respectively. Except for one outlier, all distributions show less than 6% difference of the median from the mean, and less than 10% difference of the standard deviation from the median absolute deviation. The highest difference is found for functional similarity *simGIC/fsMax*, and the lowest differences are observed for *simRes/fsBMM*. Both functional similarity measures are shown in Supplementary Figure S6, which provides several examples of score distributions by visualizing them as violin plots with overlaid boxplots. Since these differences are considerably mild, we do not expect a big impact when using modified z-scores in normalization and therefore remain with the better known standard z-scores. Lastly, we want to point out that since the distributions are non-normal, z-scores should no longer be interpreted quantitatively as mentioned at the beginning of this section.

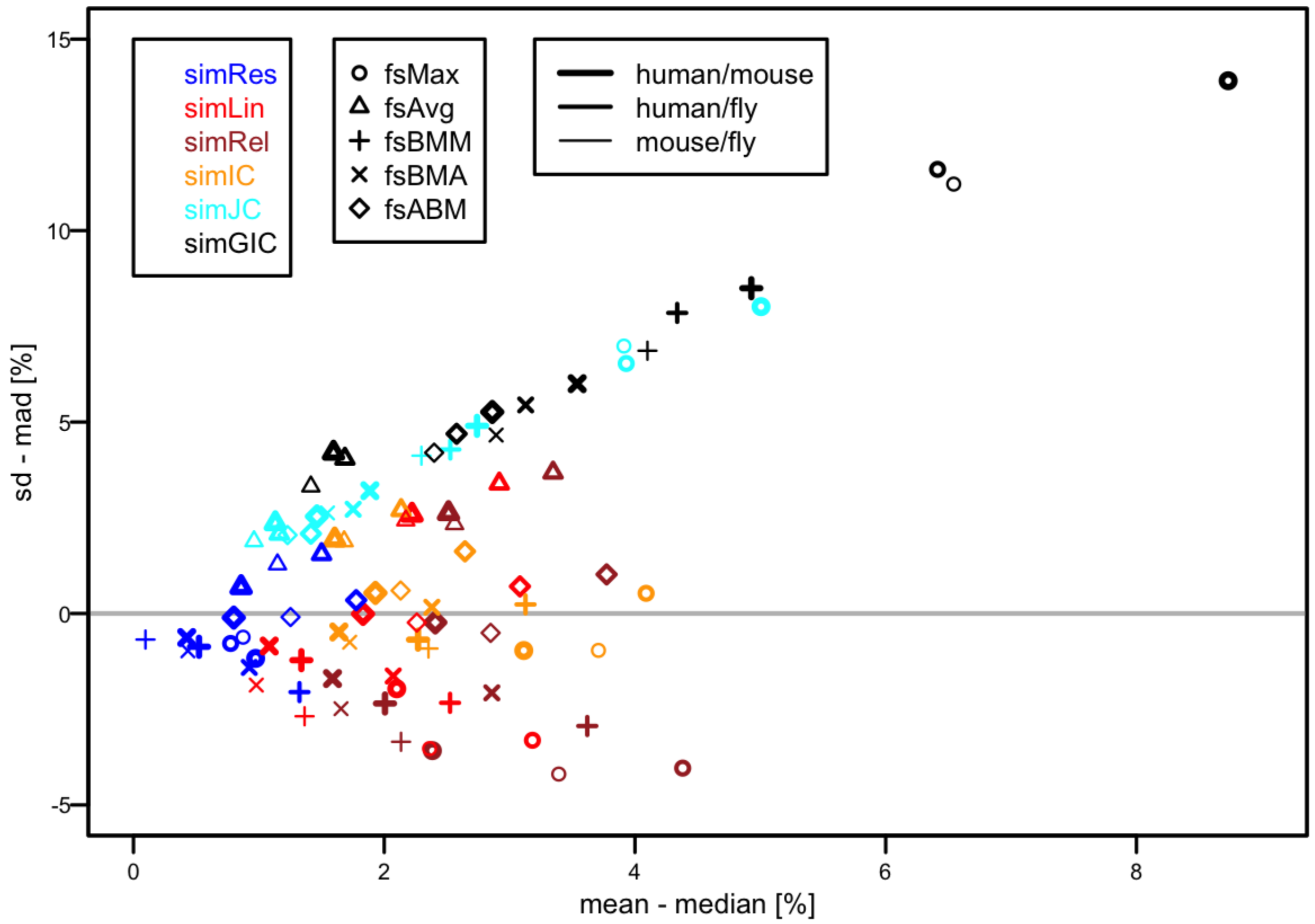

Supplementary Figure S5. Scatter plot of percentage difference of the centrality and spread measures used for z-score and modified z-score calculation. Percentages are based on the maximum value a functional similarity can achieve. This is one (1.0) for all FS measures that do not involve SS measure *simRes*, as this is an unbound measure. For measures based on *simRes,* we have chosen the highest observed value from the respective orthologous gene pairs. We note that these theoretical limits have also been observed in almost all gene pair sets, and therefore are suitable normalization constants. Points are colored by SS with shapes coding for mixing strategies, and line thickness representing gene pair sets. We use the following abbreviations: sd, standard deviation; mad, median absolute deviation.

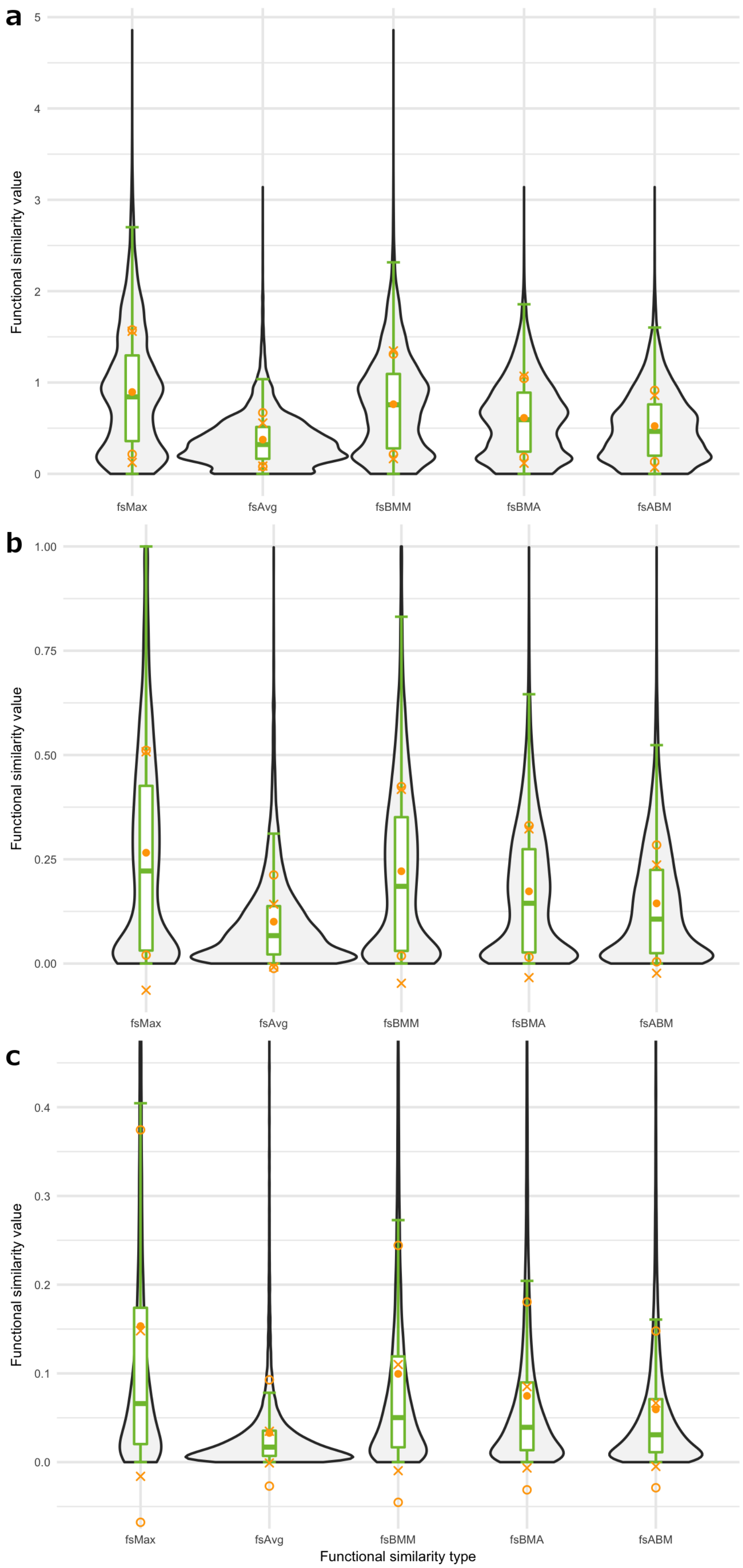
a
b
c
Functional similarity value
Functional similarity type
0
1
2
3
4
5
0.00
0.25
0.50
0.75
1.00
0.0
0.1
0.2
0.3
0.4
fsMax
fsAvg
fsBMM
fsBMA
fsABM

Supplementary Figure S6. (previous page) Violin plots and boxplots for selected distributions. Each distribution contains 50000 score values. In the boxplot, the median is shown as a green horizontal bar. One unit of median absolute deviation (mad) above and below the median are represented as yellow crosses. The distribution mean is indicated by a filled yellow point, and yellow circles are placed one unit of standard deviation below and above the mean. Boxplot whiskers extend by 1.5 times the interquartile range. Outliers are omitted. **a.** Distributions of FS scores calculated for mouse/fly random gene pairs using *simResnik* SS measure. These distributions show the smallest differences for the centrality and spread measures used for standard and modified z-score calculation. Functional similarity measure *simRes*/*fsBMM* is found to have the smallest differences: in the plot, the median and the mean are almost aligned, and units of sd and mad are also nearly overlapping. **b.** Typically observed differences, as an example we show human/fly gene pairs scored with *simRel*. Means and medians are somewhat apart, and units of sd and mad differ by some percent. **c.** Most extreme cases involve use of SS measure *simGIC*. The plot shows the FS score distribution of human/mouse gene pairs. It focuses on the high density region, and therefore omits FS values higher than 0.45. Here, we observe stronger differences, especially for the most extreme case *simGIC*/*fsMax*.

Error Rates and Confidence Intervals

We have computed 99% confidence intervals of the mean error rates based on the results of 225 repeatedly generated data sets containing the orthologous cases and an equally sized set of random controls (see main text, section “Benchmarking”). Supplementary Figure S7b shows a representative magnification of Figure 2a including 99% confidence intervals of the mean. The confidence intervals are very small, so that it can be concluded that the error rates shown in our figures are highly reliable. (See Supplementary Data File S1 for a comprehensive list of raw data.)

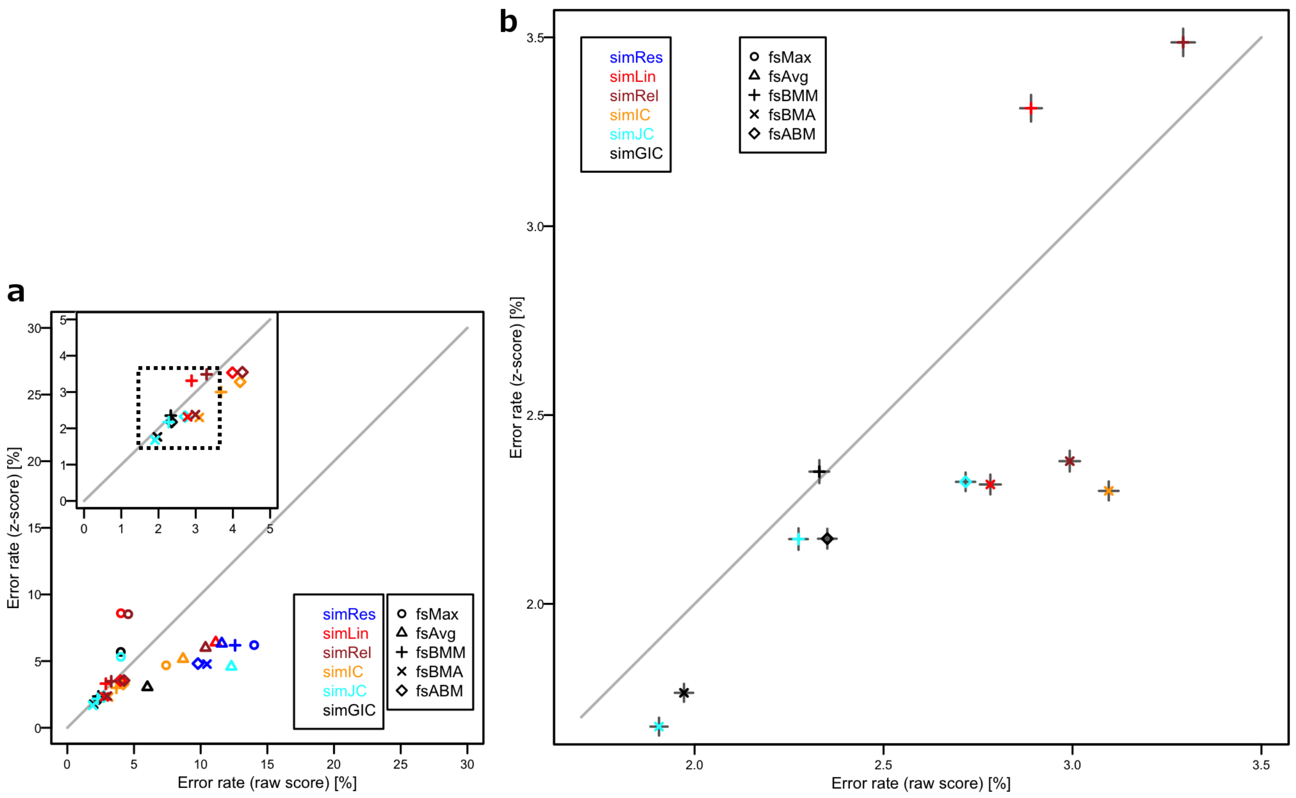

Supplementary Figure S7. Error rate scatter plots with confidence intervals comparing raw and z-scores for different FS scores based on IEA$^{(+)}$ annotations in BP ontology for human/mouse orthologues. **a.** This panel corresponds to Figure 2a from the main text. The dashed box in the magnification subplot indicates the plotting area used in panel (b) of this plot. **b.** Highly magnified region from panel (a) with 99% confidence intervals of the mean error rates shown in gray below the respective symbol. Confidence intervals are very small (typically in the range of tenth-percents) and underline the robustness of the reported error rates.

Frela Performance

We have tested runtimes of Frela on a 2.3GHz AMD Opteron 8356 processor with 32GB of RAM. We have generated random pairs of human proteins from the BP ontology, including annotations with IEA evidence codes. Proteins were randomly drawn from a pool of 93,806 annotated human proteins (see Table 1). We have chosen BP ontology, as this is the richest annotation corpus, with on average 3.7 annotations per protein. In the Supplementary Table S1 below we present the time in seconds to compute FS scores based on different SS and MS combinations for 10,000, 100,000, and 1,000,000 random protein pairs. Execution time depends mainly on the SS measure used, and all SS measures involving most informative common ancestor calculations (*simRes, simLin, simRel, simIC,* and *simJC*) have similar execution times. Semantic similarity measure *simGIC* performs more graph operations and takes about twice as long. Execution time is linear with the number of protein pairs compared. Time measurements have been taken on a running Frela server.

Supplementary Table S1. Execution time of Frela for various dataset sizes.

10,000 random protein pairs calculation time (seconds) - BP						
	**fsAvg**	**fsMax**	**fsBMM**	**fsABM**	**fsBMA**	**Average**
**simRes**	9	9	9	10	9	9.2
**simLin**	10	10	11	11	11	10.6
**simRel**	10	10	11	11	11	10.6
**simGIC**	23	22	23	23	23	22.8
**simIC**	10	10	11	11	11	10.6
**simJC**	10	10	11	10	11	10.4

100,000 random protein pairs calculation time (seconds) - BP						
	**fsAvg**	**fsMax**	**fsBMM**	**fsABM**	**fsBMA**	**Average**
**simRes**	85	85	93	92	93	89.6
**simLin**	96	96	106	104	104	101.2
**simRel**	97	97	106	106	107	102.6
**simGIC**	224	224	232	233	233	229.2
**simIC**	97	98	108	107	107	103.4
**simJC**	97	97	106	106	105	102.2

1,000,000 random protein pairs calculation time (seconds) - BP						
	**fsAvg**	**fsMax**	**fsBMM**	**fsABM**	**fsBMA**	**Average**
**simRes**	922	913	984	980	983	956.4
**simLin**	1034	1023	1120	1113	1114	1080.8
**simRel**	1031	1029	1128	1124	1123	1087
**simGIC**	2437	2424	2511	2382	2204	2391.6
**simIC**	1046	1042	1141	1133	1134	1099.2
**simJC**	1034	1037	1127	1128	1126	1090.4
